# Metabolic Impact of MKP-2 Upregulation in Obesity Promotes Insulin Resistance and Fatty Liver Disease

**DOI:** 10.3390/nu14122475

**Published:** 2022-06-15

**Authors:** Savanie Fernando, Jacob Sellers, Shauri Smith, Sarayu Bhogoju, Sadie Junkins, Morgan Welch, Orion Willoughby, Nabin Ghimire, Cassandra Secunda, Marina Barmanova, Sean C. Kumer, Kisuk Min, Ahmed Lawan

**Affiliations:** 1 Department of Biological Sciences, University of Alabama in Huntsville, Huntsville, AL 35899, USA; ysf0001@uah.edu (S.F.); jas0103@uah.edu (J.S.); shaurismith@gmail.com (S.S.); smj0024@uah.edu (S.J.); mmw0025@uah.edu (M.W.); orionw@vt.edu (O.W.); ng0063@uah.edu (N.G.); cs0244@uah.edu (C.S.); 2 Department of Chemical and Materials Engineering, University of Alabama in Huntsville, Huntsville, AL 35899, USA; sb0213@uah.edu; 3 Department of Human Nutrition, Foods and Exercise-Molecular and Cellular Science, Virginia Tech, Blacksburg, VA 24060, USA; 4 Department of Internal Medicine, Gastroenterology and Liver Center, University of Kansas Medical Center, Kansas City, MO 66160, USA; mbarmanova@kumc.edu; 5 Department of Surgery, School of Medicine, Transplantation and HPB Surgery Division, University of Kansas Medical Center, Kansas City, MO 66160, USA; seankumer@kumc.edu; 6 Department of Kinesiology, University of Texas at El Paso, El Paso, TX 79968, USA; kmin@utep.edu

**Keywords:** obesity, mitogen-activated protein kinases, protein tyrosine phosphatase, lipid and glucose metabolism, insulin resistance, fatty liver disease

## Abstract

The mechanisms connecting obesity with type 2 diabetes, insulin resistance, nonalcoholic fatty liver disease, and cardiovascular diseases remain incompletely understood. The function of MAPK phosphatase-2 (MKP-2), a type 1 dual-specific phosphatase (DUSP) in whole-body metabolism, and how this contributes to the development of diet-induced obesity, type 2 diabetes (T2D), and insulin resistance is largely unknown. We investigated the physiological contribution of MKP-2 in whole-body metabolism and whether MKP-2 is altered in obesity and human fatty liver disease using MKP-2 knockout mice models and human liver tissue derived from fatty liver disease patients. We demonstrate that, for the first time, MKP-2 expression was upregulated in liver tissue in humans with obesity and fatty liver disease and in insulin-responsive tissues in mice with obesity. MKP-2-deficient mice have enhanced p38 MAPK, JNK, and ERK activities in insulin-responsive tissues compared with wild-type mice. MKP-2 deficiency in mice protects against diet-induced obesity and hepatic steatosis and was accompanied by improved glucose homeostasis and insulin sensitivity. *Mkp-2*^−/−^ mice are resistant to diet-induced obesity owing to reduced food intake and associated lower respiratory exchange ratio. This was associated with enhanced circulating insulin-like growth factor-1 (IGF-1) and stromal cell-derived factor 1 (SDF-1) levels in *Mkp-2*^−/−^ mice. PTEN, a negative regulator of Akt, was downregulated in livers of *Mkp-2*^−/−^ mice, resulting in enhanced Akt activity consistent with increased insulin sensitivity. These studies identify a novel role for MKP-2 in the regulation of systemic metabolism and pathophysiology of obesity-induced insulin resistance and fatty liver disease.

## 1. Introduction

Obesity predisposes to the development of cardiovascular disease, type 2 diabetes (T2D), nonalcoholic fatty liver disease (NAFLD), and increased risk of insulin resistance and even some cancers [1,2]. The mechanisms connecting obesity with these diseases remain incompletely understood. Mitogen-activated protein kinases (MAPKs) are physiological regulators of metabolic homeostasis, and MAPK dysfunction promotes metabolic disease [3,4]. However, the regulatory mechanisms of MAPKs in metabolism remains under investigation. 

Mammalian MAPKs comprise three major sub-groups, and these include the extracellular signal-regulated kinases (ERK1/2), c-Jun NH_2_-terminal kinases (JNK1/2/3), and p38 MAPKs [5,6,7]. Obesity and obesity-induced inflammatory state activates the stress-responsive MAPKs, p38 MAPK, and JNK [8,9,10,11]. Muscle-specific JNK1 deletion mice displayed improved insulin sensitivity and increased glucose turnover in response to feeding of chow or high-fat diet (HFD) [12]. Activation of p38 MAPK increases hepatic gluconeogenesis and inhibits fat storage by reducing hepatic lipogenesis and stimulating fatty acid oxidation [13,14]. Collectively, these data showed that both JNK and p38 MAPK are important mediators of metabolic signaling, which regulates insulin resistance and energy metabolism.

The MAPKs are inactivated by mitogen-activated protein kinase phosphatase (MKPs) through dephosphorylation [15]. The MKPs comprise a family of 10 catalytically active enzymes that dephosphorylate MAPKs [15,16]. Members of the MKP family have been shown to play diverse roles in metabolism. For instance, MKP-1-deficient mice were resistant to diet-induced obesity [17], and skeletal muscle-specific MKP-1 deletion recapitulates the phenotype of global knockout [18]; MKP-3 regulates hepatic gluconeogenesis [19] and increases MKP-4 expression in adipose tissue of *db/db* mice [20]. MKP-2, also known as dual-specificity phosphatase 4 (DUSP4), is an inducible phosphatase known to be upregulated in response to growth factors, hormones, and lipopolysaccharide (LPS) [21] The understanding of MKP-2 function has resulted in the discovery of novel roles for MKP-2 in the regulation of sepsis, infection, and cell-cycle progression that are distinct from those of other MKPs [22,23,24,25]. One study showed that MKP-2 is a negative regulator of JNK and p38 MAPK in macrophages and that it inhibits the expression of proinflammatory cytokines in response to lipopolysaccharide (LPS) [22]. Another study suggests that MKP-2 regulates macrophage–adipocyte interaction [26]. However, the physiological function of MKP-2 in metabolism remains largely unknown. 

In this study, we revealed that MKP-2 expression was upregulated in liver tissue in humans with obesity and fatty liver disease and in insulin-responsive tissues in mice with obesity. MKP-2-deficient mice have enhanced p38 MAPK, JNK, and ERK activities in insulin-responsive tissues compared with wild-type mice. We found that MKP-2-deficient mice were protected against obesity and development of hepatic steatosis and insulin resistance following HFD feeding. These results suggest that MKP-2 plays a major role in the development of obesity, insulin resistance, and nonalcoholic fatty liver disease. 

## 2. Materials and Methods

### 2.1. Reagents, Antibodies and Immunoblotting

All reagents were purchased from standard chemical vendors. The following antibodies were used; phospho-p38 MAPK (#9215S), phospho-JNK1/2 (#4668S), phospho-ERK1/2 (#9101S), p38 MAPK (#9228S), JNK (#3708S), ERK1/2 (9102S), PTEN (#9559S), p-Akt (#9271), Akt (#2966S), and Beta actin (#8457S), were obtained from cell signaling technology. MKP-2 (#610850) was obtained from BD Biosciences. Liver, skeletal muscle, and adipose tissues from *Mkp-2*^+/+^ and *Mkp-2*^−/−^ mice were isolated and processed and immunoblotted as described [3].

### 2.2. Animal and Human Studies

#### 2.2.1. Animal Studies

The University of Alabama Institutional Animal Care and Use Committee approved all animal studies. The MKP-2 wild-type (*Mkp-2*^+/+^) and MKP-2 knockout (*Mkp-2*^−/−^) mice were kindly provided by Robin Plevin, University of Strathclyde, United Kingdom. Mice lacking MKP-2 have been genetically characterized previously [22,24]. For in vivo studies under normal and diet-induced obesity conditions, three-week-old male and female mice were maintained on either a custom high-fat (60% kcal) purified rodent diet DN 112252 (Dyets, Inc.,Bethlehem, PA, USA) or chow diet or chow diet (Lab Supply, Nothlake, TX, USA) for 16 to 24 weeks. The ingredients and nutrient composition of these diets are shown in Table 1 and Table 2 for 16 to 24 weeks. 

#### 2.2.2. Human Liver Samples 

Liver biopsy samples from normal and obese NASH individuals (males ~70% and females ~30%) between the ages of 30 and 68 years were used. The specimens were provided by the University of Kansas Liver Center Biorepository. Normal individuals had a BMI ~25 kg/m^2^ with no evidence of infiltrates or fibrosis and obese NASH (BMI ~30 kg/m^2^); clinically steatohepatitis with lobular inflammation and NAFLD; and clinically steatosis without NASH (BMI ~30 kg/m^2^).

### 2.3. Metabolic Measurements

Glucose tolerance tests (GTTs) were performed on male and female *Mkp-2*^+/+^ and *Mkp-2*^−/−^ mice fed either chow or HFD for 16 to 24 weeks. Mice were fasted overnight for 16 h followed by intraperitoneally (i.p.) injection of glucose (2 g/kg). Blood glucose levels were measured at time points 0, 15, 30, 60, 90, and 120 min. For insulin tolerance tests (ITTs), mice were fasted for 5 h and were injected (i.p.) with 0.75 mU/g human insulin (Humlin R; Elly and Company, Indianapolis, IN, USA). The blood glucose levels were measured at time points 0, 15, 30, 60, 90, and 120 minutes. Plasma insulin levels were measured using ultra-sensitivity mouse insulin Elisa kit (Crystal Chem, Elk Grove Village, IL, USA; Catalog #90080).

Conscious male chow-fed *Mkp-2*^+/+^ and *Mkp-2*^−/−^ mice aged between 8 to 12 weeks old were individually housed under controlled temperature (23 °C) and lighting (12 h light, 12 h dark cycle, lights on at 0700 h) with free access to food and water. Mice were adapted for one week and kept in metabolic cages (Promethion, Sable Systems, Las Vegas, NV, USA) for one week, and the food and water intake, energy expenditure, respiratory exchange ratio (RER), and physical activity were measured. Data were analyzed using regression analysis using ANCOVA.

### 2.4. Cell Culture and Transient Transfections

Mouse embryonic fibroblasts (MEFs) derived from *Mkp-2*^+/+^ and *Mkp-2*^−/−^ mice were cultured as described [3] and transfected with pcDNA3-FLAG PTEN (Cat.#78777; Addgene). MEFs were serum-starved overnight before stimulated with insulin (100 nM) for 1 h. At the end of stimulation, MEFs were lysed using RIPA buffer as described [3]. Plasmids were transfected with Lipofectamine 3000 (Invitrogen, Carlsbad, CA, USA) according to the manufacturer’s instructions.

### 2.5. RNA Extraction and Real-Time PCR Analysis

Tissues derived from *Mkp-2*^+/+^ and *Mkp-2*^−/−^ mice were used to isolate total RNA using RNeasy kit (Qiagen, Germantown, MD, USA) according to the manufacturer’s instructions and as described [3]. Real-time quantitative PCR was performed as described [3], with the Applied Biosystems 7500 Fast RT-PCR system and TaqMan and SYBR Green gene expression master mix with the following primer pairs: *Srebf2*, 5′-GCAGCAACGGGACCATTCT-3′ and 3′-CCCCATGACTAAGTCCTTCAACT-5′; 18S, 5′-ACCGCAGCTAGGAATAATGGA-3′ and 3′-GCCTCAGTTCCGAAAACCA-5′; *Srebf1c*, 5′-ATCTCCTAGAGCGAGCGTTG and 3′-TATTTAGCAACTGCAGATATCCAAG; CD36, 5′-ATGGGCTGTGATCGGAACTG-3′ and 3′-TTTGCCACGTCATCTGGGTTT-5′; CCL2, 5′-TTAAAAACCTGGATCGGAACCAA-3′ and 3′-GCATTAGCTTCAGATTTACGGGT-5′; and CPT1α, 5′-TGTCAAAGATACCGTGAGCAG-3′ and 5′-GCCCACCAGGATTTTAGCTT-3′.

All relative gene expression levels were analyzed using the ΔCt method and normalized to 18S. TaqMan primers and gene expression master mix from Applied Biosystems were used for FASN and quantitation.

### 2.6. Serum Cytokine Levels Measurements

Serum cytokine levels derived from HFD-fed *Mkp-2*^+/+^ and *Mkp-2*^−/−^ mice were analyzed using mouse cytokine antibody array, which contains 96 different anti-cytokine antibodies, including negative and positive controls (AAM-CYT-1000-4, RayBiotech Life, Inc., Peachtree Corners, GA, USA).

### 2.7. Histological Analysis of Tissue Sections

Tissues were isolated from chow and HFD-fed male *Mkp-2*^+/+^ and *Mkp-2*^−/−^ mice and then fixed in 4% paraformaldehyde in PBS and processed for paraffin sections and stained with hematoxylin and eosin. Cryostat sections of livers were processed for Oil Red O staining as described [3].

### 2.8. Statistical Analysis

All data are presented as mean ± SEM. Differences between groups were compared using Student’s unpaired two-tailed *t*-test or one- and two-way analysis of variance (ANOVA). A post hoc test was performed using Bonferroni’s multiple comparisons using Prism 9 statistical software (GraphPad Software, La Jolla, CA, USA). A value of *p* < 0.05 was considered statistically significant. Gender was not considered a factor in the statistical analysis. Indirect calorimetry was analyzed using CaIR, a web-based analysis tool [27].

### 2.9. Data and Resource Availability

All data generated or analyzed during this study are included in the published article (and its online supplementary files). The human samples analyzed during the current study were obtained from University of Kansas Liver Center Biorepository.

## 3. Results

### 3.1. Upregulation of MKP-2 Expression in Human and Mice Livers with Obesity and Fatty Liver Disease

**Figure 1:**
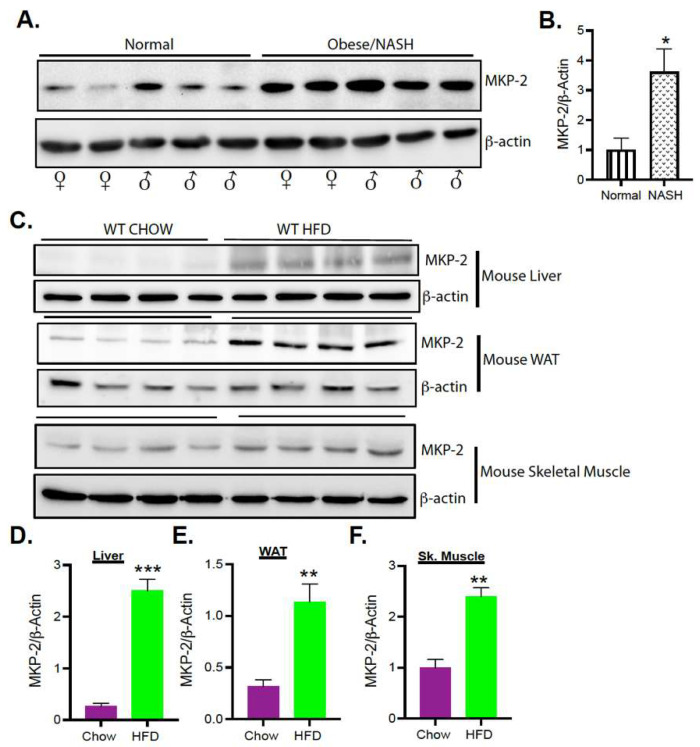
Upregulation of MKP-2 Expression in Human and Mice Livers with Obesity and Fatty Liver Disease. Liver tissue lysates from male and female normal (BMI ~25 kg/m^2^) and obese NASH (BMI ~30 kg/m^2^) human subjects (N = 7/group) (**A**,**B**). Liver, skeletal muscle, and white adipose tissue lysates from wild-type chow and HFD-fed mice (N = 4–6) (**C**–**F**) were analyzed by immunoblotting. Representative immunoblots were quantitated by densitometry for the levels of MKP-2 and β-actin. Results represent the mean ± SEM; * *p* < 0.05, ** *p* < 0.01, *** *p* < 0.0001 as determined by Student’s *t*-test.

To investigate the role of MKP-2 in obesity and development of NAFLD and nonalcoholic steatohepatitis (NASH), we determined the protein expression levels of MKP-2 in livers of male and female normal and obese NASH humans. Interestingly, in the livers of obese NASH subjects, we found for the first time that the levels of MKP-2 protein expression were significantly enhanced (>3-fold; *p* < 0.05) as compared with normal subjects (Figure 1A,B). Furthermore, we determined the protein expression levels of MKP-2 in obese mice across major metabolic tissues. In the liver of wild-type, high-fat diet (HFD)-fed mice, we found that the expression levels of MKP-2 protein were significantly elevated (~5-fold; *p* < 0.001) as compared with wild-type chow-fed livers (Figure 1C, upper panel and D). Similarly, we also found that MKP-2 protein expression levels were significantly elevated (~3-fold; *p* < 0.01) in white adipose tissue (WAT) of HFD-fed wild-type mice (Figure 1C, middle panel and E) as compared with wild-type chow-fed WAT. In addition, we found that the expression levels of MKP-2 protein were significantly elevated in skeletal muscle in HFD-fed wild-type mice (~2.5-fold; *p* < 0.001.) as compared with chow-fed skeletal muscles. (Figure 1C, lower panel and F). These results demonstrate that MKP-2 is upregulated in liver tissue in humans with obesity and fatty liver disease and in insulin-responsive tissues in mice with obesity and may contribute to the development of obesity and NAFLD/NASH. We hypothesize that overexpression of MKP-2 following overnutrition promotes the development of obesity and fatty liver disease.

### 3.2. MAPK Phosphorylation in Human NASH Livers

We assessed the phosphorylation status of JNK, p38 MAPK, and ERK in the livers of normal and NASH subjects. In livers of obese NASH human subjects, the phosphorylation levels of JNK were increased in NASH livers (Figure S1A upper panel and B) compared with livers of normal subjects, but these were not statistically significant. However, no differences were observed in p38 MAPK or ERK phosphorylation levels in the livers of NASH compared with normal subjects (Figure S1A middle and lower panels, and C,D). These results suggest that the activity of JNK, p38 MAPK, and ERK were comparable in obese NASH and normal subjects.

### 3.3. Enhanced MAPK Phosphorylation in MKP-2-Deficient Mice

**Figure 2:**
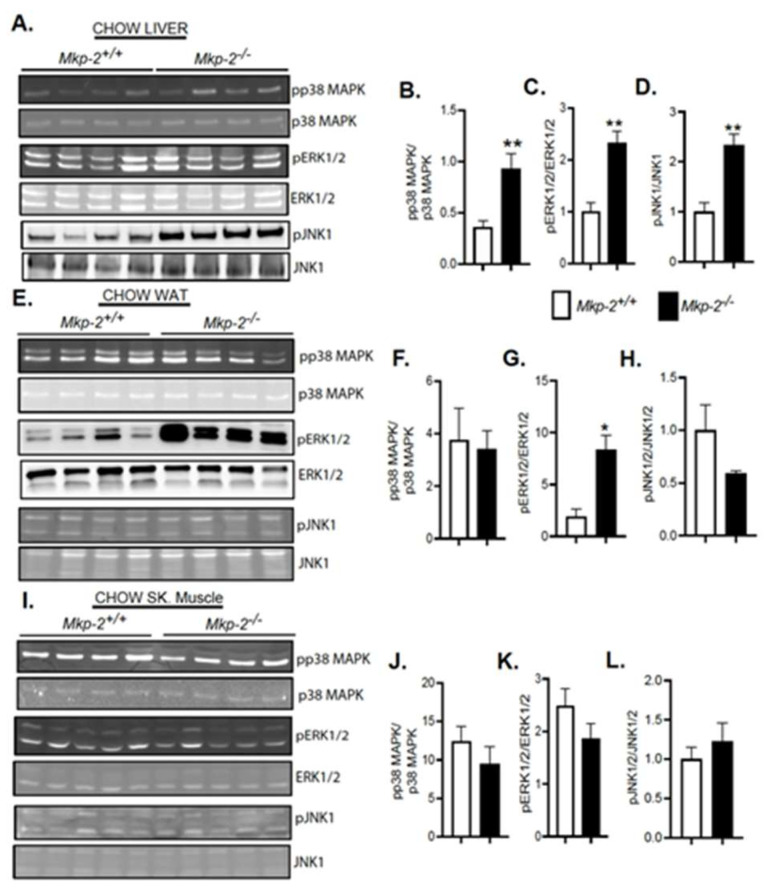
Enhanced MAPK Phosphorylation in Chow- and HFD-fed MKP-2-Deficient Mice. Liver tissue lysates from liver, skeletal muscle, and white adipose tissue lysates from chow- and HFD-fed mice. *Mkp-2^+/+^* and *Mkp-2^−/−^* were analyzed by immunoblotting (N = 4–6). Representative immunoblots were quantitated by densitometry for phospho-p38 MAPK/p38 ((**A**) upper panel and (**B**)), phospho-JNK1/2/JNK1/2 ((**A**) lower panel and (**C**)), and phospho-ERK1/2ERK1/2 ((**A**) middle panel (**D**)) in chow liver; phospho-p38 MAPK/p38 MAPK ((**E**) upper panel and (**F**)), phospho-ERK1/2ERK1/2 ((**E**) middle panel (**G**)), and phospho-JNK1/2/JNK1/2 ((**E**) lower panel and (**H**)) in chow WAT; phospho-p38 MAPK/p38 MAPK ((**I**) upper panel and (**J**)), phospho-ERK1/2ERK1/2 ((**I**) middle panel (**K**)), and phospho-JNK1/2/JNK1/2 ((**I**) lower panel and (**L**)) in chow skeletal muscle. Results represent the mean ± SEM; * *p* < 0.05, ** *p* < 0.01 as determined by Student’s *t*-test.

Given the role of MAPKs in metabolic control, understanding the function of MKP-2 in their regulation is very important. We determined the phosphorylation status of p38 MAPK, JNK, and ERK across major metabolic tissues. Consistent with lack of MKP-2, we found that MKP-2 global knockout (*Mkp-2*^−/−^) mice exhibited significantly enhanced p38 MAPK (Figure 2A, upper panel and B), ERK (Figure 2A, middle panel and C), and JNK (Figure 2A, lower panel and D) phosphorylation in the liver under chow-fed conditions compared with wild-type (*Mkp-2*^+/+^) mice. In white adipose tissue (WAT), chow-fed *Mkp-2*^−/−^ mice exhibited significantly enhanced ERK (Figure 2E, middle panel and G) phosphorylation compared with wild-type (*Mkp-2*^+/+^) mice. However, no differences were observed in p38 MAPK (Figure 2E, upper panel and F) or JNK phosphorylation levels (Figure 2E, lower panel and H) in the WAT of chow-fed *Mkp-2*^−/−^ compared with *Mkp-2*^+/+^ mice. No differences were observed in p38 MAPK (Figure 2I, upper panel and J), ERK (Figure 2I, middle panel and K), or JNK (Figure 2I, lower panel and L) phosphorylation in the skeletal muscle of chow-fed *Mkp-2*^−/−^ and *Mkp-2*^+/+^ mice. Consequently, lack of MKP-2 expression results in the corresponding upregulation of p38 MAPK in the liver but not WAT or skeletal muscle and JNK and ERK in the liver and WAT but not in skeletal muscle.

### 3.4. Resistance to Diet-Induced Obesity in MKP-2-Deficient Mice

**Figure 3:**
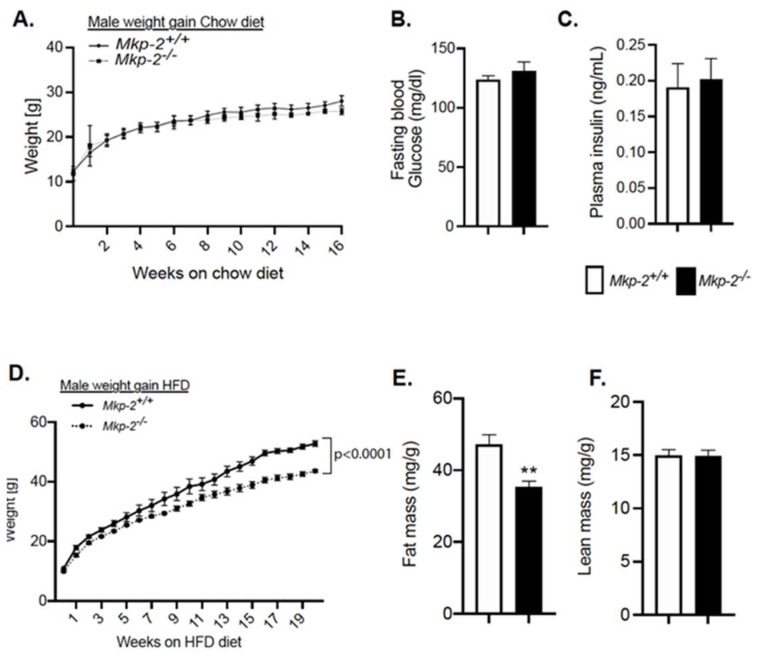
Resistance to Diet-Induced Obesity and Insulin Sensitivity in Male MKP-2-Deficient Mice. (**A**) Weight curves of chow-fed *Mkp-2^+/+^* and *Mkp-2^−/−^* mice for 16 weeks; (**B**) fasting blood glucose; (**C**) plasma insulin; (**D**) weight curves of HFD-fed *Mkp-2^+/+^* and *Mkp-2^−/−^* mice for 24 weeks; (**E**) fat mass; and (**F**) lean mass (N = 10 mice/genotype). Results represent the mean ± SEM; ** *p* < 0.01, as determined by Student’s *t*-test or in (**A**,**D**) by analysis of variance (ANOVA) with Bonferroni’s post-test for multiple comparisons. Open bars, *Mkp-2^+/+^* mice; closed bars, *Mkp-2^−/−^* mice.

*Mkp-2*^+/+^ and *Mkp-2*^−/−^ mice were bred and, from three weeks of age, were fed either chow diet or HFD for a period of 16 to 24 weeks. Male *Mkp-2*^−/−^ mice fed a chow diet exhibited comparable weight gain as compared with *Mkp-2*^+/+^ mice (Figure 3A). There was no difference in fasting blood glucose or plasma insulin between chow-fed male *Mkp-2*^+/+^ and *Mkp-2*^−/−^ mice (Figure 3B,C). Male *Mkp-2*^−/−^ mice fed a HFD had a significantly (*p* = 0.0001) reduced rate of weight gain as compared with *Mkp-2*^+/+^ mice (Figure 3D). By 17 weeks of high-fat feeding, male *Mkp-2*^−/−^ mice weighed ~22% less as compared with *Mkp-2*^+/+^ mice (Figure 3D). No differences were observed in skeletal muscle histological sections liver, adipose or skeletal muscle tissue weight between chow-fed *Mkp-2*^+/+^ and *Mkp-2*^−/−^ mice (Figure S2A–E). Consistent with reduced fat mass in HFD-fed (Figure 3E), white adipose tissue histological presentation showed that the size of adipocytes of *Mkp-2*^−/−^ mice is smaller than that of *Mkp-2*^+/+^ mice (Figure S3). No difference in lean mass between HFD-fed male *Mkp-2*^+/+^ and *Mkp-2*^−/−^ mice was discovered (Figure 3B,C). To further investigate the involvement of MKP-2 in reduced fat content, we assessed the phosphorylation of p38 MAPK, ERK, and JNK in WAT derived from HFD-fed *Mkp-2*^+/+^ and *Mkp-2*^−/−^ mice. The results indicated that JNK and ERK phosphorylation were significantly enhanced in *Mkp-2*^−/−^ compared with *Mkp-2*^+/+^ mice (Figure S4A middle and lower panels, and C,D). However, no differences were observed in p38 MAPK phosphorylation (Figure S4A, upper panel and B) in the WAT of *Mkp-2*^−/−^ compared with *Mkp-2*^+/+^ mice. Female *Mkp-2*^−/−^ mice fed a chow diet exhibited reduced weight gain as compared with *Mkp-2*^+/+^ mice (Figure S5A). However, female *Mkp-2*^−/−^ mice fed a HFD diet exhibited comparable weight gain compared with *Mkp-2*^+/+^ mice (Figure S5E). Liver weights were also similar (Figure S5D). These results demonstrate that HFD-fed female *Mkp-2*^−/−^ mice were not resistant to diet-induced obesity. Together, these data demonstrate that loss of MKP-2 protects against the development of diet-induced obesity and insulin resistance in male mice.

### 3.5. Reduced Food Intake and RER in MKP-2-Deficient Mice

**Figure 4:**
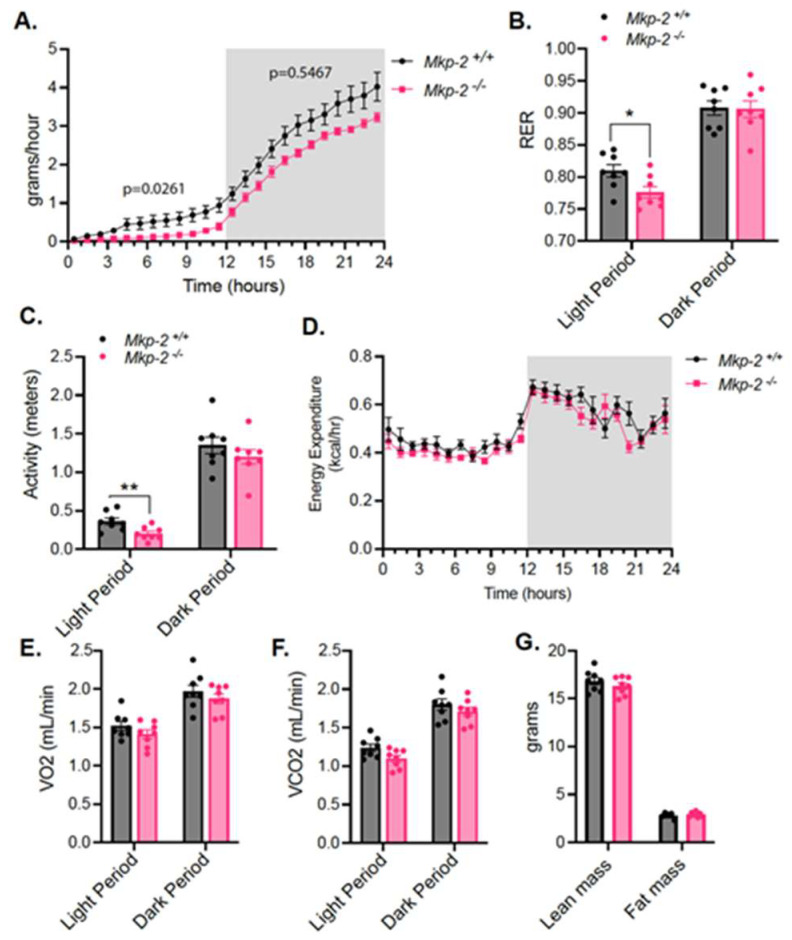
Reduced food intake and RER in MKP-2-deficient mice. (**A**–**G**) Chow-fed *Mkp-2^+/+^* and *Mkp-2^−/−^* mice were subjected to indirect calorimetry. (**A**) Food intake; (**B**) respiratory exchange ratio; (**C**) locomotor activity; (**D**) energy expenditure; (**E**) oxygen consumption; (**F**) carbon dioxide production; and (**G**) lean and fat mass (N = 8 per genotype). Data represent mean ± SEM * *p* < 0.05, ** *p* < 0.01, as determined by Student’s *t*-test or by ANCOVA analysis.

In order to determine the physiological mechanism accounting for the resistance to weight gain in *Mkp-2*^−/−^ mice, we performed metabolic calorimetry. Chow-fed *Mkp-2*^−/−^ mice exhibit significantly reduced food intake and RER in the light cycle compared with *Mkp-2*^+/+^ mice (Figure 4A,B). These findings are consistent with the fact that *Mkp-2*^−/−^ mice eat less; then, their RER would be expected to be lower, indicating that *Mkp-2*^−/−^ mice burn more lipids from their fat stores compared with *Mkp-2*^+/+^ mice. In addition, the activity levels of *Mkp-2*^−/−^ mice were also significantly reduced compared with *Mkp-2*^+/+^ mice during the light cycle (Figure 4C). However, we did not observe any differences in energy expenditure between *Mkp-2*^+/+^ and *Mkp-2*^−/−^ mice (Figure 4D–F). Similarly, *Mkp-2*^−/−^ and *Mkp-2*^+/+^ mice exhibit comparable total body lean and fat mass (Figure 4G). These data support the hypothesis that the *Mkp-2*^−/−^ mice are resistant to diet-induced obesity owing to reduced food intake that is associated with lower RER. This suggests that the *Mkp-2*^−/−^ mice burn lipids from their fat stores instead of eating. Interestingly, the *Mkp-2*^−/−^ mice did not compensate by eating more during the dark phase. During the dark phase, the *Mkp-2*^−/−^ mice ate slightly less than the *Mkp-2*^+/+^ mice, but it did not reach significance (Figure 4A,B). Together, these data suggest that MKP-2 deficiency results in reduced food intake and RER, consistent with observed resistance to weight gain on a high-fat diet.

### 3.6. Protection from the Development of Hepatic Steatosis in MKP-2-Deficient Mice

**Figure 5:**
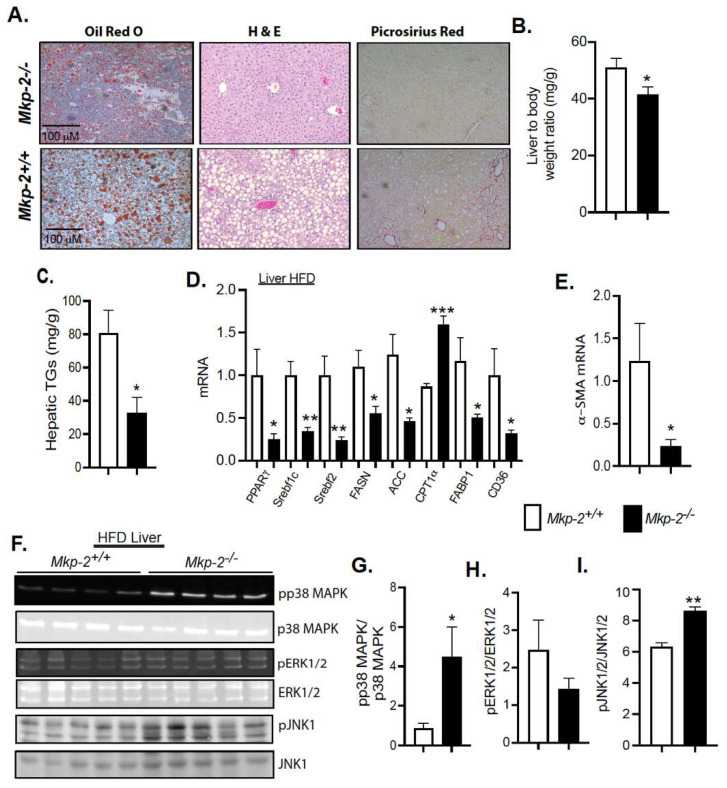
Protection from the Development of Hepatic Steatosis in HFD-fed Male MKP-2-Deficient Mice. (**A**) Representative hematoxylin and eosin staining, Oil Red O and Picrosirius red staining of liver sections from HFD-fed *Mkp-2^−/−^* mice ((**A**), upper panel) and *Mkp-2^+/+^* mice ((**A**), lower panel) mice for 24 weeks (N = 5 mice/genotype). (**B**) Liver weights; (**C**) hepatic TGs; (**D**) hepatic mRNA expression of PPARγ, Srebf1c, Srebf2, FASN, ACC, CPT1α, FABP1 and CD36; and (**E**) alpha smooth muscle actin. Representative immunoblots were quantitated by densitometry for phospho-p38 MAPK/p38 ((**F**) upper panel and (**G**)), phospho-JNK1/2/JNK1/2 ((**F**) lower panel and (**I**)), and phospho-ERK1/2ERK1/2 ((**F**) middle panel (**H**)) in HFD livers. Results represent the mean ± SEM; * *p* < 0.05, ** *p* < 0.01, *** *p* < 0.0001 as determined by Student’s *t*-test or in (**D**) by analysis of variance (ANOVA) with Bonferroni’s post-test for multiple comparisons. Open bars, *Mkp-2^+/+^* mice; closed bars, *Mkp-2^−/−^* mice.

Consequent to HFD feeding for 24 weeks, lipid content were markedly decreased in the livers of male MKP-2-deficient mice as compared with *Mkp-2*^+/+^, mice, suggesting that MKP-2 deficiency prevents the development of hepatic steatosis (Figure 5A, upper panel *Mkp-2*^−/−^, lower panel *Mkp-2*^+/+^). No differences were observed histologically or in tissue weights in the liver, skeletal muscle, WAT, and brown adipose tissue (BAT) under chow-fed conditions (Figure S2A–E). Consistent with resistance to hepatic steatosis, *Mkp-2*^−/−^ mice exhibited a marked reduction in liver weight (Figure 5B) and hepatic triglycerides (TGs) (Figure 5C) compared with *Mkp-2*^+/+^ mice. Furthermore, *Mkp-2*^−/−^ mice exhibited reduced fat mass compared with *Mkp-2*^+/+^ mice (Figure 4B). Consistent with protection from fatty liver, the expression of mRNAs for PPARγ, sterol regulatory element-binding protein 1c (Srebf1c), and Srebf2 were significantly reduced in the livers of *Mkp-2*^−/−^ mice (Figure 5D). Under HFD feeding, important factors promoting lipogenesis in hepatocytes, fatty acid synthase (FASN), and acetylcoA carboxylase (ACC) were significantly reduced in the *Mkp-2*^−/−^ mice compared *Mkp-2*^+/+^ mice (Figure 5D). Furthermore, hepatic mRNA expression of carnitine palmitoyltransferase 1, a key rate-limiting enzyme in the fatty acid β-oxidation pathway, was significantly enhanced in *Mkp-2*^−/−^ mice compared with *Mkp-2*^+/+^ mice (Figure 5D). In addition, the mRNA levels of fatty acid uptake gene, FABP1, were significantly reduced in the livers of *Mkp-2*^−/−^ mice compared with *Mkp-2*^+/+^ mice (Figure 5D). Fatty acid translocase (CD36) plays a major role in fatty acid uptake in many metabolic tissues, including the liver [28]. We analyzed the mRNA expression of CD36 in the livers of *Mkp-2*^−/−^ mice. In HFD-fed *Mkp-2*^−/−^ livers, we found that the expression levels of CD36 were significantly reduced (~70%; *p* < 0.05) compared with *Mkp-2*^+/+^ mice (Figure 5D). These results suggest that *Mkp-2*^−/−^ livers exhibit CD36-dependent reduction in fatty acid uptake, thereby lowering the lipid content and conferring protection from the development of hepatic steatosis. Reduced liver damage was observed in HFD-fed MKP-2-deficient mice as evident in decreased hepatic fibrosis as assessed by picrosirius red staining (Figure 5A, upper panel *Mkp-2*^+/+^, lower panel *Mkp-2*^−/−^). Furthermore, analysis of markers of fibrosis demonstrated that *Mkp-2*^−/−^ mice exhibited significantly reduced gene expression of alpha smooth muscle actin (α-SMA) compared with *Mkp-2*^+/+^ mice (Figure 5E). To further investigate the involvement of MKP-2 in reduced hepatic steatosis, we assessed the phosphorylation of p38 MAPK, ERK, and JNK in liver derived from HFD-fed *Mkp-2*^+/+^ and *Mkp-2*^−/−^ mice. We found that p38 MAPK and JNK phosphorylation were significantly enhanced in *Mkp-2*^−/−^ compared with *Mkp-2*^+/+^ mice (Figure 5F upper and lower panels and G,I). However, no differences were observed in ERK phosphorylation (Figure 5F, middle panel and H) in the liver of *Mkp-2*^−/−^ compared with *Mkp-2*^+/+^ mice. Together, these results indicate that MKP-2-deficient mice fed a HFD were protected from development of hepatic steatosis by reducing lipogenesis and fatty uptake in hepatocytes. It also suggests that in obesity, MKP-2 upregulation impairs hepatic fatty acid β-oxidation, thereby promoting accumulation of fat in the liver.

### 3.7. Glucose Tolerance and Insulin Sensitivity in Chow and HFD-Fed MKP-2-Deficient Mice

**Figure 6:**
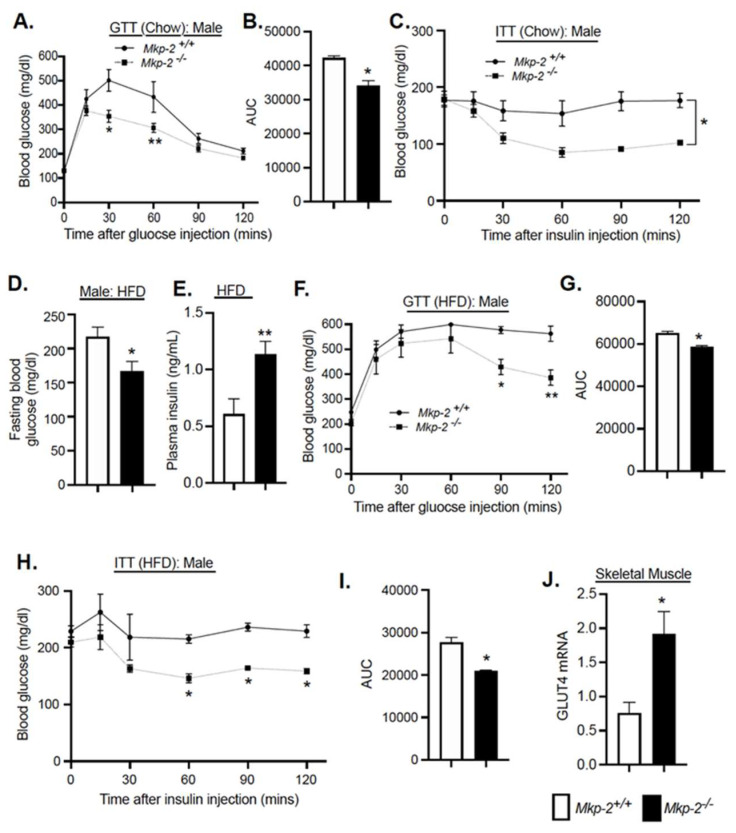
Glucose Tolerance and Insulin Sensitivity in Male Chow-fed MKP-2-Deficient Mice. Plasma glucose concentration during GTTs (**A**), AUC (**B**), and ITTs (**C**); fasting blood glucose (**D**), plasma insulin (**E**), and plasma glucose concentration during GTTs (**F**), AUC (**G**), and ITTs (**H**); AUC (**I**) and GLUT4 mRNA expression in skeletal muscle (**J**) from HFD-fed *Mkp-2^+/+^* and *Mkp-2^−/−^* mice (N = 5–10/genotype). Results represent the mean ± SEM; * *p* < 0.05, ** *p* < 0.01, as determined by analysis of variance (ANOVA) with Bonferroni’s post-test for multiple comparisons. Open bars, *Mkp-2^+/+^* mice; closed bars, *Mkp-2^−/−^* mice.

To examine the effects of MKP-2 deficiency on systemic clearance of glucose in *Mkp-2*^−/−^ mice, we performed glucose tolerance tests (GTTs). GTTs were performed with male and female *Mkp-2*^+/+^ and *Mkp-2*^−/−^ mice fed either chow or HFD. Mice were fasted overnight for 16 h, followed by an injection (i.p.) of glucose at 2 g/kg body weight. Blood glucose was measured as described in methods. The data showed no difference in fasting blood glucose or plasma insulin between chow-fed male *Mkp-2*^+/+^ and *Mkp-2*^−/−^ mice (Figure 3B,C). Interestingly, in chow-fed conditions, blood glucose concentration reduced significantly in the male *Mkp-2*^−/−^ mice at 30 and 60 min after glucose injection as compared with *Mkp-2*^+/+^ mice (Figure 6A). This was also reflected by the area under the curve (Figure 6B). To examine the effects of i.p. administration of insulin on systemic glucose clearance and assess insulin sensitivity, we performed insulin tolerance tests (ITTs). *Mkp-2*^+/+^ and *Mkp-2*^−/−^ mice were fasted for 5 h and injected (i.p.) with 0.75 mU/g human insulin. ITTs demonstrated that changes in blood glucose level in chow-fed male*Mkp-2*^−/−^ mice were significantly lower than those in the *Mkp-2*^+/+^ mice after insulin injection (Figure 6C). In HFD-fed conditions, fasting blood glucose in HFD-fed male *Mkp-2*^−/−^ mice were significantly reduced compared with *Mkp-2*^+/+^ mice (Figure 6D). Plasma insulin levels were significantly increased in HFD-fed *Mkp-2*^−/−^ mice compared with *Mkp-2*^+/+^ mice (Figure 6E), suggesting that *Mkp-2*^−/−^ mice exhibit physiological hyperinsulinemia. The GTTs demonstrate that blood glucose concentration reduced significantly in the male *Mkp-2*^−/−^ mice at 90 and 120 min after glucose injection compared with *Mkp-2*^+/+^ mice (Figure 6F). This was also reflected by the area under the curve (Figure 6G). ITTs demonstrated that changes in blood glucose level in the HFD-fed male *Mkp-2*^−/−^ mice were significantly lower than those in the *Mkp-2*^+/+^ mice at 60, 90, and 120 min after insulin injection (Figure 6H). This was also reflected by the area under the curve (Figure 6I). No differences were observed in fasting blood glucose (Figure S5B,C), GTTs, and ITTs in chow- and HFD-fed female *Mkp-2*^+/+^ and *Mkp-2*^−/−^ mice (Figure S5G–J). Furthermore, we found that the expression levels of MKP-2 protein was slightly increased in the HFD-fed livers of female mice as compared with wild-type chow-fed livers, but this was not statistically significant (Figure S5K,L). These results demonstrate that male *Mkp-2*^−/−^ mice exhibit improved ability to reduce glucose levels and improve insulin sensitivity, suggesting that MKP-2 contributes to the maintenance of glucose homeostasis. 

To investigate how MKP-2-deficient mice exhibit glucose tolerance, we examined the potential mechanisms associated with glucose metabolism. GLUT4 is key to glucose uptake in skeletal muscle [29]. We found that the expression levels of GLUT4 in skeletal muscles of chow-fed *Mkp-2*^−/−^ mice were significantly enhanced compared with *Mkp-2*^+/+^ mice (Figure 6J). These results suggest that *Mkp-2*^−/−^ mice display increased basal whole-body glucose disposal, thereby contributing to the improved glucose tolerance. 

### 3.8. Enhanced Akt Signaling in MKP-2-Deficient Mice

**Figure 7:**
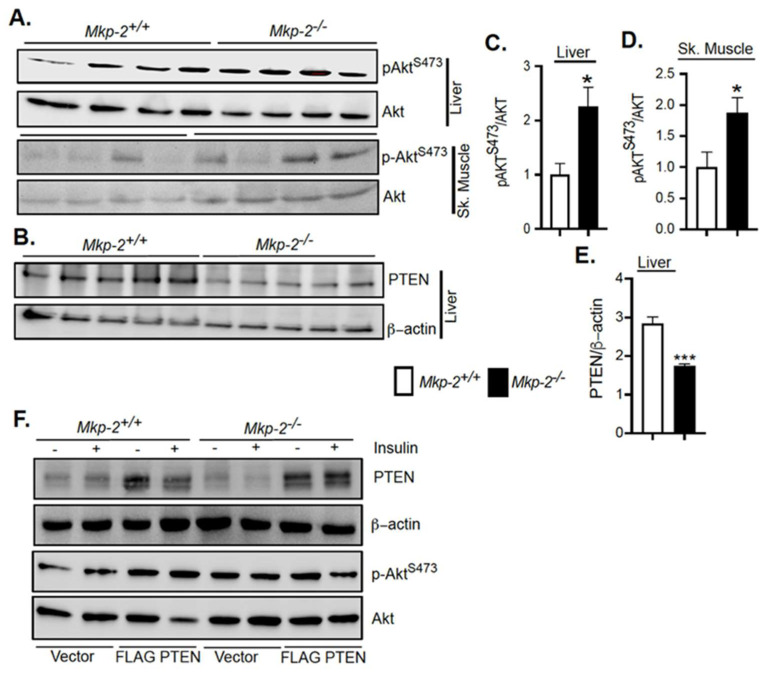
Enhanced Akt signaling in MKP-2-Deficient Mice. Liver and skeletal muscle lysates from overnight fasted insulin stimulated (i.p.) HFD-fed (5 weeks) *Mkp-2^+/+^* and *Mkp-2^−/−^* mice were analyzed by immunoblotting (N = 4–6 mice/genotype). Representative immunoblots were quantitated by densitometry for phospho-Akt/Akt, liver (**A**,**C**), and skeletal muscle (**A**,**D**) and PTEN and β-actin (**B**,**E**). (**F**) Serum-starved MEFs were transfected with vector or FLAG PTEN followed by insulin (100 nM) stimulation for 1 h. Results represent the mean ± SEM; * *p* < 0.05, *** *p* < 0.0001, as determined by Student’s *t*-test. Open bars, *Mkp-2^+/+^* mice; closed bars, *Mkp-2^−/−^* mice.

**Figure 8:**
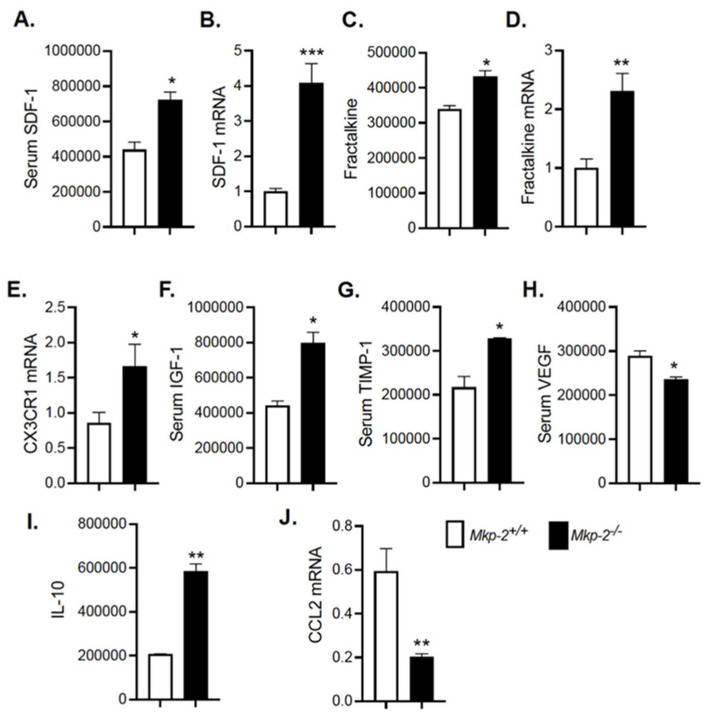
Modulation of Cytokines/Chemokines and Growth factor Secretion in HFD-fed Male MKP-2-Deficient mice. Serum protein levels of secreted cytokines from HFD-fed *Mkp-2^+/+^* and *Mkp-2^−/−^* mice for 24 weeks (N = 4/genotype). (**A**) SDF-1, (**B**) hepatic SDF-1 mRNA, (**C**) fractalkine, (**D**) hepatic fractalkine mRNA, and (**E**) its receptor CX3CR1, (**F**) IGF-1, (**G**) TIMP-1, (**H**) VEGF, IL-10 (**I**), and (**J**) CCL-2. Results represent the mean ± SEM; * *p* < 0.05, ** *p* < 0.01, *** *p* < 0.0001, as determined by Student’s *t*-test. Open bars, *Mkp-2^+/+^* mice; closed bars, *Mkp-2^−/−^* mice.

To establish the molecular basis for the improved glucose homeostasis and increased insulin sensitivity in *Mkp-2*^−/−^ mice (Figure 6), we assessed the phosphorylation status of Akt in the liver, skeletal muscle, and adipose tissue. Remarkably, in the liver (Figure 7A,C) and skeletal muscles (Figure 7A,D) of *Mkp-2*^−/−^ mice, phosphorylation of Akt^S473^ was significantly enhanced as compared with *Mkp-2*^+/+^ mice. However, in adipose tissue, no significant differences in Akt phosphorylation were observed between *Mkp-2*^−/−^ mice and *Mkp-2*^+/+^ mice (Figure S6A,B). Akt is found downstream of IGF-1R signaling pathway [30], and this is consistent with enhanced serum levels of IGF-1 and improved ability to reduce glucose levels in *Mkp-2*^−/−^ mice (Figure 8F). These results demonstrate that MKP-2 plays a role in negatively regulating Akt pathway in the liver and skeletal muscle. This observation that MKP-2 negatively regulates Akt encouraged us to examine the mechanism of MKP-2/Akt cross-talk. We tested the hypothesis that Akt signaling could be affected by changes in the expression levels of phosphatase and tensin homolog (PTEN), which negatively regulates Akt [21]. When we measured the expression levels of PTEN in the livers of *Mkp-2*^−/−^ mice, we found significantly decreased levels of PTEN in these mice as compared with *Mkp-2*^+/+^ mice (Figure 7B,E), suggesting that in the liver, MKP-2 negatively regulates Akt activity by regulating PTEN expression. To examine the mechanism that reduced PTEN expression in *Mkp-2*^−/−^ mice that plays a major role for the elevated level of Akt phosphorylation, we investigated the MKP-2/PTEN/Akt phosphorylation axis in mouse embryonic fibroblasts (MEFs). Consistent with the in vivo results, we observed enhanced basal levels of Akt phosphorylation *Mkp-2*^−/−^ MEFs compared with *Mkp-2*^+/+^ MEFs (Figure 7F, lane 1 vs. lane 5). Furthermore, in response to insulin stimulation, we found enhanced Akt phosphorylation in *Mkp-2*^−/−^ MEFs compared with *Mkp-2*^+/+^ MEFs (Figure 7F, lane 2 vs. lane 6). Interestingly, when PTEN was overexpressed in *Mkp-2*^−/−^ MEFs, and this restored the level of Akt phosphorylation comparable to *Mkp-2*^+/+^ MEFs (Figure 7F, lanes 3 and vs. lanes 7 and 8). These data suggest that the improved insulin sensitivity observed in *Mkp-2*^−/−^ mice results from enhanced phosphorylation of Akt that is partly due to reduced PTEN expression.

### 3.9. Modulation of Cytokines/Chemokines and Growth Factor Secretion in HFD-Fed MKP-2-Deficient Mice

In order to evaluate the possibility that MKP-2 regulates the expression and secretion of cytokines, cell–cell signaling proteins that systemically control the progression of obesity, and development of insulin resistance and hepatic steatosis, we performed a mouse cytokine antibody array to analyze for the levels of 96 mouse proteins in blood serum of HFD-fed *Mkp-2*^+/+^ and *Mkp-2*^−/−^ mice. Densitometric analysis showed changes in *Mkp-2*^−/−^ mice protein secretion profile. We found that a select group of molecules were overrepresented. Among those that showed statistically elevated levels in *Mkp-2*^−/−^ mice compared with *Mkp-2*^+/+^ mice were stromal cell-derived factor (SDF-1) (Figure 8A) and insulin-like growth factor-1 (IGF-1) (Figure 8F). Others were fractalkine (FKN) (Figure 8C), TIMP-1 (Figure 8G), and IL-10 (Figure 8I). Most interesting were the presence of insulin-like growth factor-1 (IGF-1), stromal cell-derived factor 1 (SDF-1), fractalkine (FKN), and IL-10. To verify the cytokine antibody array results, quantitative RT-PCR was performed in livers of *Mkp-2*^+/+^ and *Mkp-2*^−/−^ mice. We found an increase of ~4-fold in SDF-1 and ~2-fold in fractalkine mRNA expression in the livers of *Mkp-2*^−/−^ mice compared to *Mkp-2*^+/+^ mice (Figure 8B,D). We also found FKN cognate receptor, CX3CR1, was significantly upregulated in *Mkp-2*^−/−^ mice (Figure 8E). Studies showed that in vivo administration of FKN improved glucose tolerance [31]. Moreover, SDF-1 KO mice exhibit glucose intolerance and insulin resistance [32]. These results indicate that the expression of these cytokines were enhanced in the livers of MKP-2-deficient mice, which is consistent with the insulin sensitivity observed in *Mkp-2*^−/−^ mice. IGF-1 is essential for normal insulin sensitivity, and dysregulation of IGF-1 synthesis results in the development of insulin resistance [33]. Similarly, increased TIMP-1 expression reduced liver fibrosis [34]. These studies are consistent with enhanced serum IGF-1 and TIMP-1 expression in *Mkp-2*^−/−^ mice. Moreover, adipose tissue overexpression of VEGF protects against diet-induced obesity and insulin resistance [35], and MKP-2 deficient mice exhibit reduced serum VEGF levels (Figure 8H). Obesity and metabolic syndrome promote the development of inflammation. We found that the serum levels of the anti-inflammatory cytokine IL-10 were significantly elevated in *Mkp-2*^−/−^ mice (Figure 8I). Furthermore, we found a significant decrease (~3-fold; *p* < 0.01) in the mRNA expression of a pro-inflammatory cytokine, C-C chemokine ligand, CCL2, also known as monocyte chemoattractant protein 1 (MCP-1), in the livers of *Mkp-2*^−/−^ mice compared to *Mkp-2*^+/+^ mice (Figure 8J). Collectively, our data suggest that MKP-2 promotes IGF-1, SDF-1, FKN, and IL-10 signaling to contribute to development of insulin resistance, NAFLD, fibrosis, and inflammation.

## 4. Discussion

In this study, we show for the first time that MKP-2 protein levels were increased in the liver of obese humans with NASH. Consistent with this, we show that MKP-2 was increased in its protein levels in the liver, skeletal muscle and white adipose tissues in mice fed an HFD. We show that MKP-2-deficient mice exhibit enhanced p38 MAPK, JNK, and ERK activities in insulin-responsive tissues. We found enhanced phosphorylation of p38 MAPK in the liver of chow- and HFD-fed MKP-2-deficient mice but not skeletal muscle or WAT. Additionally, in the liver, the phosphorylation JNK and ERK were increased. In WAT, we found enhanced JNK and ERK phosphorylation. These results show that MKP-2 is a major physiological regulator of the activities of p38 MAPK, JNK, and ERK in major insulin-responsive tissues and suggest that increased activity of these MAPKs mediate the metabolic function of MKP-2. 

Many studies demonstrated either enhanced activity or deficiency in each of these MAPKs play a key role in the regulation of metabolic homeostasis and potential targets for treatment of metabolic diseases [4,36,37]. Although previous studies mainly from in vitro data suggested MKP-2 does not dephosphorylate p38 MAPK [32], our data clearly demonstrate that MKP-2 regulate the activity of p38 MAPK in the liver but not in skeletal muscle or WAT. This strongly suggests that the function of MKP-2 in terms of substrate specificity in vivo may be cell-type-specific. We also observed enhanced JNK and ERK activities in MKP-2-deficient mice, and these data indicated that the observed phenotype in this model could be mediated by more than one MAPK family member. *Mkp-2*^−/−^ mice exhibit enhanced JNK activity, which is widely reported to impair glucose homeostasis. However, *Mkp-2*^−/−^ mice exhibit increased glucose homeostasis. We think that since MKP-2 is localized in the nucleus, this discrepancy could be due inactivation of the nuclear pool of JNK by MKP-2 that is different from the regulation of cytosolic pool of JNK. Our data indicate that MKP-2 at least in the liver plays a role in negatively regulating both p38 MAPK and ERK but additionally the Akt pathway. The results reported here highlight the physiological specificity within MKPs family members, indicating some differences and similarities between our data and other knockout models of MKPs. For instance, MKP-1 whole-body knockout mice were resistant to diet-induced obesity but not insulin sensitive [17], and MKP-3 regulates hepatic gluconeogenesis [19] and increased MKP-4 expression in adipose tissue of *db/db* mice [20]. 

We find that the main physiological mechanism responsible for the reduced adiposity and resistance to diet-induced obesity exhibited by the MKP-2-deficient mice was attributable to reduced food intake. Furthermore, the reduced RER in MKP-2-deficient mice demonstrate that these mice burn lipids from their fat stores instead of eating. Although many different organs are involved in food intake regulation, our data raise the fascinating possibility that MKP-2 may have direct effects on the hypothalamic neurons that regulate feeding behavior [38]. Recently, it has been shown that MKP-2 knockout mice exhibit enhanced local cerebral glucose utilization in the ventral tegmental area, a ghrelin-sensitive brain nucleus [39]. This indicate that MKP-2 is an important regulator of pathways that control lipid homeostasis. We showed PPARγ expression levels were significantly reduced in the livers of *Mkp-2*^−/−^ mice; therefore, it is possible that enhanced JNK and/or ERK activities in adipocytes of *Mkp-2*^−/−^ mice dysregulate PPARγ-dependent lipogenesis. Moreover, *Mkp-2*^−/−^ mice exhibited enhanced levels of Akt in the liver and skeletal muscle, which prompts us to discover a pathway by which MKP-2 negatively regulates AKT by antagonizing PTEN. These results suggest that loss of MKP-2 stimulates changes in the expression of the PTEN/Akt pathway to contribute to the increased insulin sensitivity. Furthermore, our cytokine antibody array data showed significantly enhanced serum IGF-1 levels in MKP-2-deficient mice, supporting the observed increased Akt singling and insulin sensitivity in MKP-2-deficient mice. Consistent with this, patients with IGF-1 gene deletion exhibit severe insulin resistance [40], and mice with IGF-1 deletion in the liver exhibit insulin resistance and administration of IGF-1 improved the insulin-resistant state [40]. Similarly, our data showed that serum SDF-1 was significantly enhanced in MKP-2-deficient mice. This is consistent with the observed MKP-2-deficient phenotype, as streptozotocin (STZ) injected SDF-1 transgenic mice exhibit improved glucose homeostasis and were protected from the development of diabetes through Akt activation in β-cells [41]. Moreover, in vivo administration of fractalkine has been shown to improve glucose tolerance and promotes β-cell function [31] consistent with increased serum fractalkine in MKP-2-deficient mice. The mechanisms by which β-cell functional plasticity regulate insulin secretion in pathological conditions are unclear, and examining tissue-specific contribution of MKP-2 could potentially identify novel pathways that regulate β-cell physiology to enhance insulin secretion.

Our data demonstrate that MKP-2 plays an important role in hepatic lipid metabolism. MKP-2-deficient mice were protected from the development of fatty liver. This is consistent with enhanced hepatic expression of CPT1α in *Mkp-2*^−/−^ mice, indicating that MKP-2 negatively regulates fatty acid oxidation in the liver. Liver-specific deletion of PPARγ improves hepatic steatosis [42]. The phenotype of MKP-2-deficient mice recapitulates in part those of PPARγ liver-specific-deficient mice, which manifest as resistance to diet-induced acquisition of fatty liver and significant reduction in levels of hepatic PPARγ. Furthermore, deletion of JNK1 in hepatocytes promotes hepatic steatosis [43], suggesting that increased activity of JNK protects against hepatic steatosis. In addition, ERK2 liver-specific knockout mice exhibit hepatic steatosis, suggesting that enhanced ERK activity prevents hepatic steatosis [44]. Studies have shown that liver-specific expression of transcriptionally active Srebf1c promotes development of fatty liver and increased visceral mass [45], and mice expressing nuclear Srebf1c developed fatty liver [46] and liver-specific Srebf2 knockout mice exhibit reduced cholesterol and fatty acid synthesis [47]. This is consistent with significantly reduced Srebf1c and Srebf2 in the livers of *Mkp-2*^−/−^ mice, suggesting that MKP-2 negatively regulates hepatic fatty acid oxidation and cholesterol metabolism. Moreover, it has been shown that Srebf1c is phosphorylated by p38 MAPK and ERK in HepG2 cells [48]. Mechanistically, MKP-2 negatively regulates Srebf2 expression by attenuating p38 MAPK pathway, suggesting its contribution to the metabolic effects of MKP-2 deficiency in fatty liver. Together, these data suggest MKP-2-deficient mice exhibit p38 MAPK-dependent impaired hepatic de novo lipogenesis. Obesity and metabolic syndrome promote the development of inflammation. Although many studies have demonstrated the regulation of immune function by MKPs, our study for the first time suggests the role of MKP-2 in the development of obesity-induced inflammation. We showed that MKP-2-deficient mice exhibit attenuated obesity-induced inflammation. Consistent with our findings, in a model of experimental autoimmune encephalomyelitis (EAE), it has been reported that MKP-2 knockout mice exhibit reduced severity of EAE [49]. These findings suggest that MKP-2 plays a vital role in the regulation of obesity-induced inflammation and could be a possible therapeutic target for the treatment of metabolic syndrome.

In summary, we demonstrate for the first time a critical role of MKP-2 in development of obesity, insulin resistance, and fatty liver disease in vivo. Our results demonstrate that MKP-2 is a major regulator of p38 MAPK, JNK, and ERK activities in insulin responsive tissues, and upregulation of MKP-2 in obesity contributes to the development of insulin resistance, fatty liver disease, and metabolic dysfunction. These observations implicate MKP-2 as a potential target for the treatment obesity and fatty liver disease and possibly other metabolic diseases. 

## Figures and Tables

**Table 1:** The ingredients and nutrient composition of high-fat diet used in the study.

Ingredient	kcal./g	g/kg	kcal./kg
Casein	3.58	200	716.00
Constarch	3.6	0	0.00
Dyetrose	3.8	125.00	475.00
Sucrose	4	68.8	275.20
Cellulose	0	50	0.00
Soybean Oil	9	25	225.00
TBHQ	0	0.005	0.00
Lard	9	245	2205.00
Salt Mix #210088	1.6	10	16.00
Dicalcium Phosphate	0	13	0.00
Calcium Carbpnate	0	5.5	0.00
Patassium Citrate H2O	0	16.5	0.00
Vitamin Mix #300050	3.92	10	39.20
L-Cystine	4	3	12.00
Choline Bitartrate	0	2	0.00
		**773.805**	**3963.400**

TBHQ: t-Butylhy droquinone.

**Table 2:** The ingredients and nutrient composition of chow diet used in the study.

Ingredient	kcal./g	g/kg	kcal./kg
Casein	3.58	200	716.00
L-Cystine	4	3	12.00
Sucrose	4	350	1400.00
Constarch	3.6	315	1134.00
Dyetrose	3.8	35	133.00
Soybean Oil	9	25	225.00
t-Butylhy droquinone	0	0.005	0.00
Lard	9	20	180.00
Cellulose	0	50	0.00
Salt Mix #210088	1.6	10	16.00
Dicalcium Phosphate	0	13	0.00
Calcium Carbpnate	0	5.5	0.00
Patassium Citrate H2O	0	16.5	0.00
Vitamin Mix #300050	3.92	10	39.20
Choline Bitartrate	0	2	0.00
		**1055.005**	**3855.200**

## Supplementary Materials

The following supporting information can be downloaded at: https://www.mdpi.com/article/10.3390/nu14122475/s1, Figure S1: MAPK Phosphorylation in Human Fatty Liver Disease; Figure S2: No difference in histology of MKP-2 deficient mice and wild type mice; Figure S3: Skeletal muscle and WAT histology of MKP-2 deficient mice and wild type mice; Figure S4: MAPK Phosphorylation in chow-fed MKP-2 Deficient Mice; Figure S5: Glucose Tolerance and Insulin Sensitivity in female Chow and HFD-fed MKP-2 Deficient Mice; Figure S6: No difference in WAT Akt phosphorylation in MKP-2 deficient mice and wild.
