# Supplementary material for: Metabolic Impact of MKP-2 Upregulation in Obesity Promotes Insulin Resistance and Fatty Liver Disease
Source: Nutrients. 2022 Jun 15;14(12):2475. doi: 10.3390/nu14122475 (PMC9228271; doi:10.3390/nu14122475)
Supplement: Supplementary file 1 [file nutrients-14-02475-s001.zip › nutrients-1702545-supplementary.pdf]

Metabolic Impact of MKP-2 Upregulation in Obesity Promotes Insulin Resistance and Fatty Liver Disease

Savanie Fernando[1], Jacob Sellers[1], Shauri Smith[1] Sarayu Bhogoju[2], Sadie Junkins[1],Marina Barmanova[4], Sean C Kumer[5], Kisuk Min[3], and Ahmed Lawan[1]*

1Department of Biological Sciences, University of Alabama in Huntsville, Huntsville,Alabama 35899, USA
2Department of Chemical and Materials Engineering, University of Alabama in Huntsville, Huntsville, Alabama 35899, USA.
3Department of Kinesiology, University of Texas at El Paso, El Paso, Texas 79968,USA
4Department of Internal Medicine, Gastroenterology and Liver Center, University of Kansas Medical Center, Kansas City, 66160, USA
5Department of Surgery, School of Medicine, Transplantation and HPB Surgery Division, University of Kansas Medical Center, Kansas City, 66160, USA

Supplementary data: Six (6) figures
SUPPLEMENATARY FIGURE LEGENDS

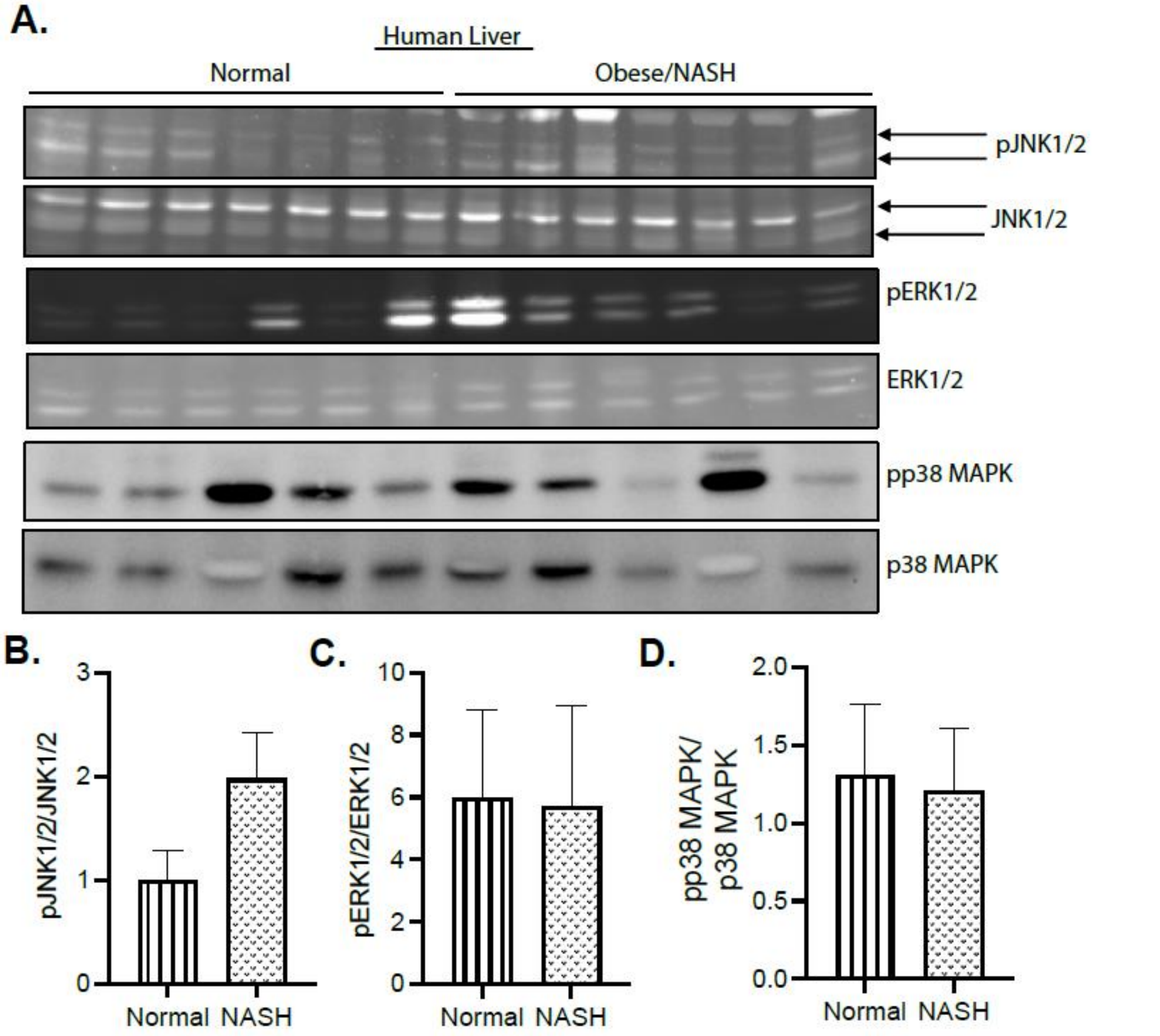

Figure S1. MAPK Phosphorylation in Human Fatty Liver Disease.

Liver tissue lysates from normal (BMI ~ 25 kg/m2) and obese NASH (BMI 30kg/m2) human subjects (n=7) were analyzed by immunoblotting. Representative immunoblots were quantitated by densitometry for phospho-JNK1/2/JNK1/2 (A and B), phospho-ERK1/2/ERK1/2 (A and C), phospho-p38 MAPK/p38 MAPK (A and D). Results represent the mean ± SEM; *; $p < 0.05$, as determined by student's t-test.

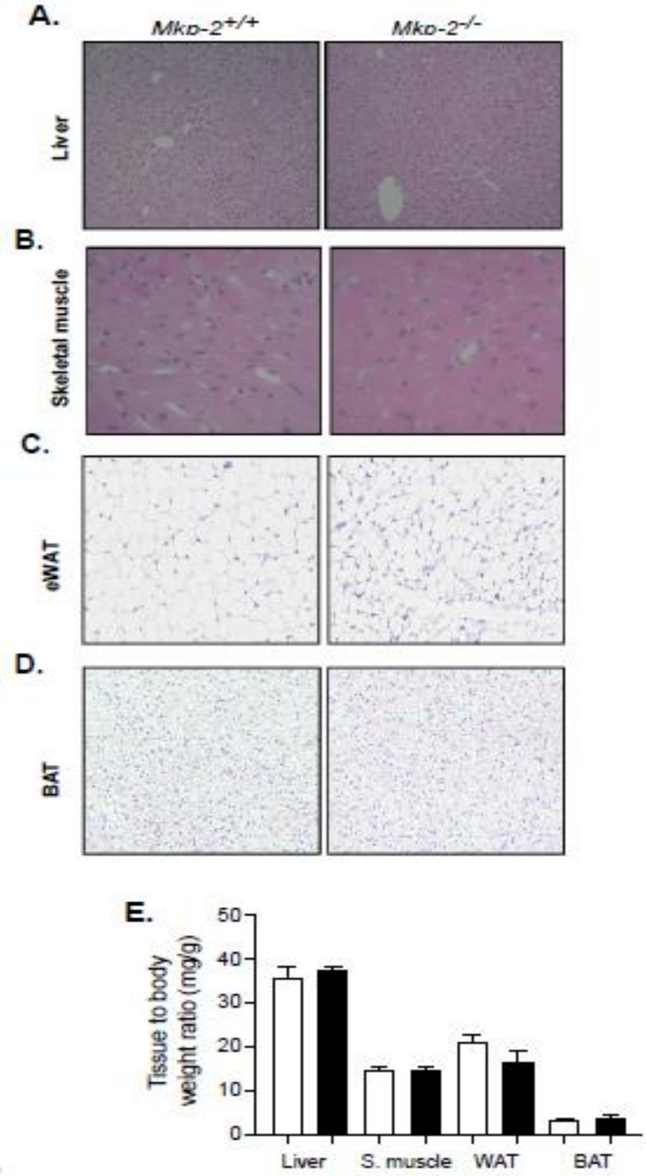

Figure S2. No difference in histology of MKP-2 deficient mice and wild type mice. Representative hematoxylin and eosin staining of liver (A), skeletal muscle (B), WAT(C) and BAT (D) sections from chow-fed Mkp-$2^{-/-}$ and Mkp-$2^{+/+}$ mice for 16 weeks (n=5 mice/genotype). (E) Tissue weights of chow-fed Mkp-$2^{-/-}$ and Mkp-$2^{+/+}$ mice for 16 weeks (n=5 mice/genotype). Open bars, Mkp-$2^{+/+}$ mice; closed bars, Mkp-$2^{-/-}$ mice.

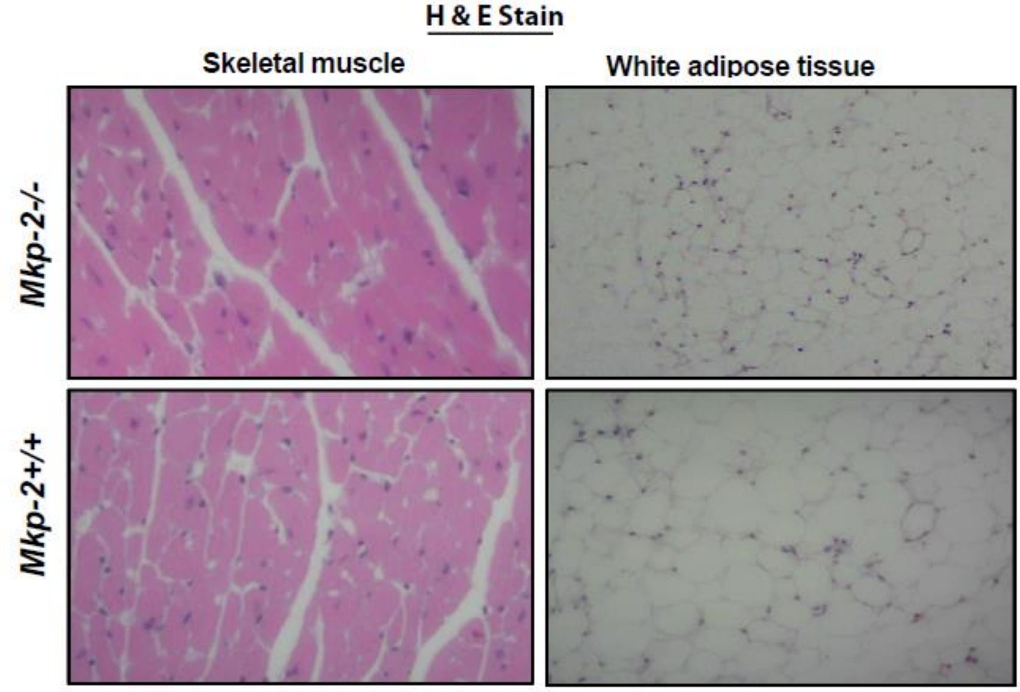

Figure S3. Skeletal muscle and WAT histology of MKP-2 deficient mice and wild type mice.

Representative hematoxylin and eosin staining of skeletal muscle and WAT sections from HFD-fed Mkp-$2^{-/-}$ and Mkp-$2^{+/+}$ mice for 24 weeks (n=5 mice/genotype).

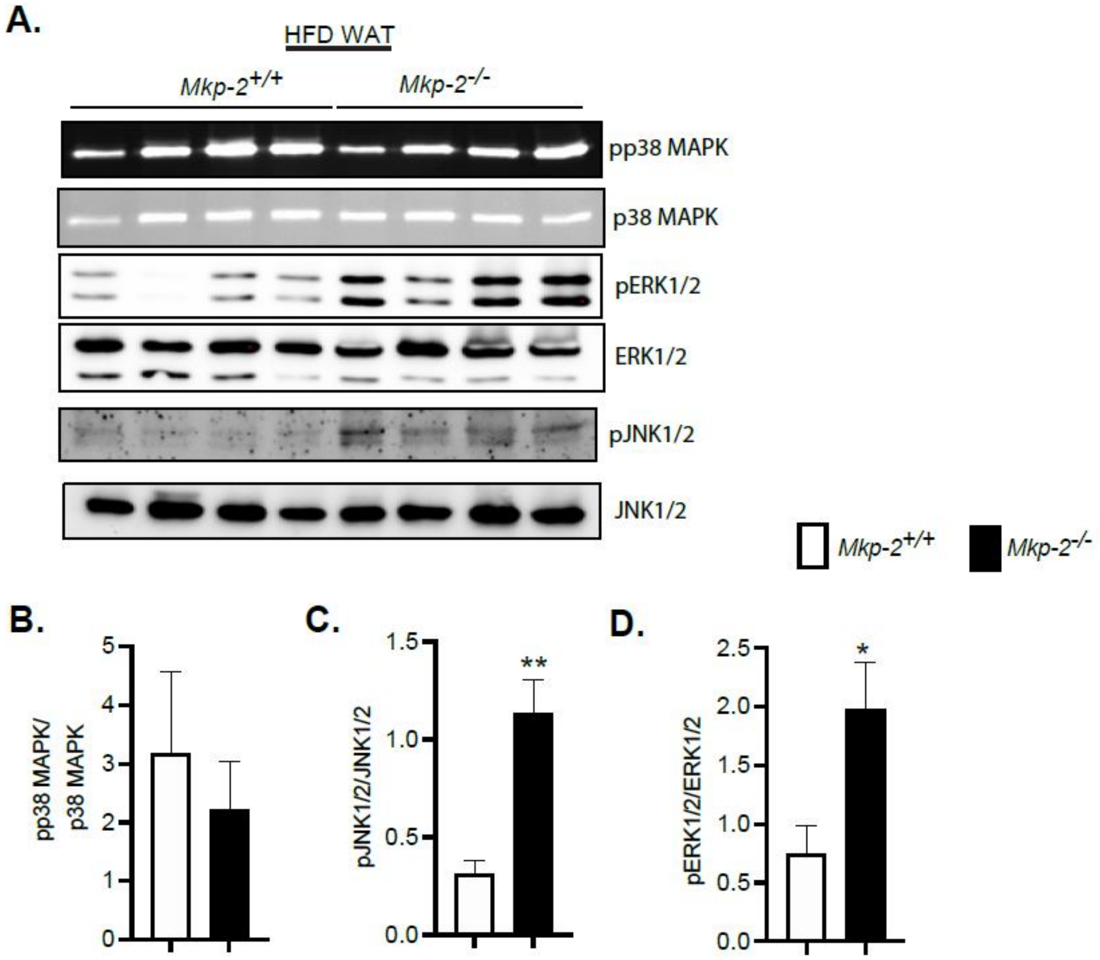

Figure S4. MAPK Phosphorylation in chow-fed MKP-2 Deficient Mice.

White adipose tissue lysates from chow-fed mice Mkp-$2^{+/+}$ and Mkp-$2^{-/-}$ were analyzed by immunoblotting (n=4-6). Representative immunoblots were quantitated by densitometry for phospho-38 MAPK/p38 MAPK (A and B), phospho-JNK1/2/JNK1/2 (A and C), phospho-ERK1/2/ERK1/2 (A and D), Results represent the mean ± SEM;*; $p < 0.05$, as determined by student's t-test.

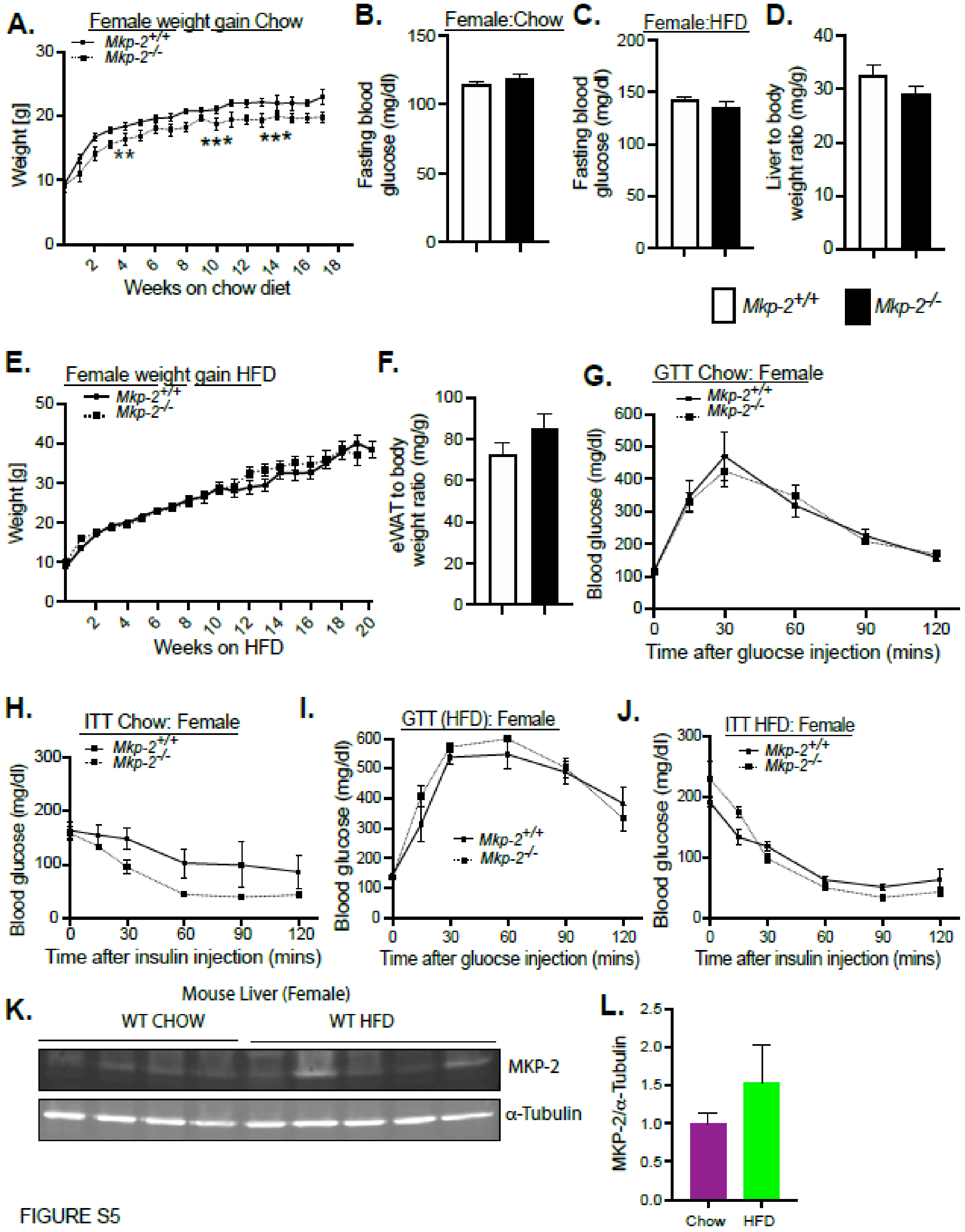

Figure S5. Glucose Tolerance and Insulin Sensitivity in female Chow and HFD-fed MKP-2 Deficient Mice.

(A)Growth curve (chow) (B) Fasting blood sugar (chow), (C) HFD; (D) Liver weight, (E) Growth curve HFD, (F) White adipose tissue weight; (G) Plasma glucose concentration during GTTs (chow), (H) ITTs (chow) (I) GTTs (HFD), (J) ITTs (HFD) Plasma glucose concentration during GTTs (B), AUC (C) and ITTs (D); GLUT4 mRNA expression in skeletal muscle (E) from Mkp-2$^{+/+}$ and Mkp-2$^{-/-}$ mice (n= 5-10/genotype). Liver tissue lysates from chow- and HFD-fed mice Mkp-2$^{+/+}$ were analyzed by immunoblotting (n=4-5). Representative immunoblots were quantitated by densitometry for

MKP-2/a-Tubulin (K and L). Results represent the mean ± SEM; *;$p < 0.05$, **; $p < 0.01$, as determined by analysis of variance (ANOVA) with Bonferroni's post-test for multiple comparisons. Open bars, Mkp-$2^{+/+}$ mice; closed bars, Mkp-$2^{-/-}$ mice.

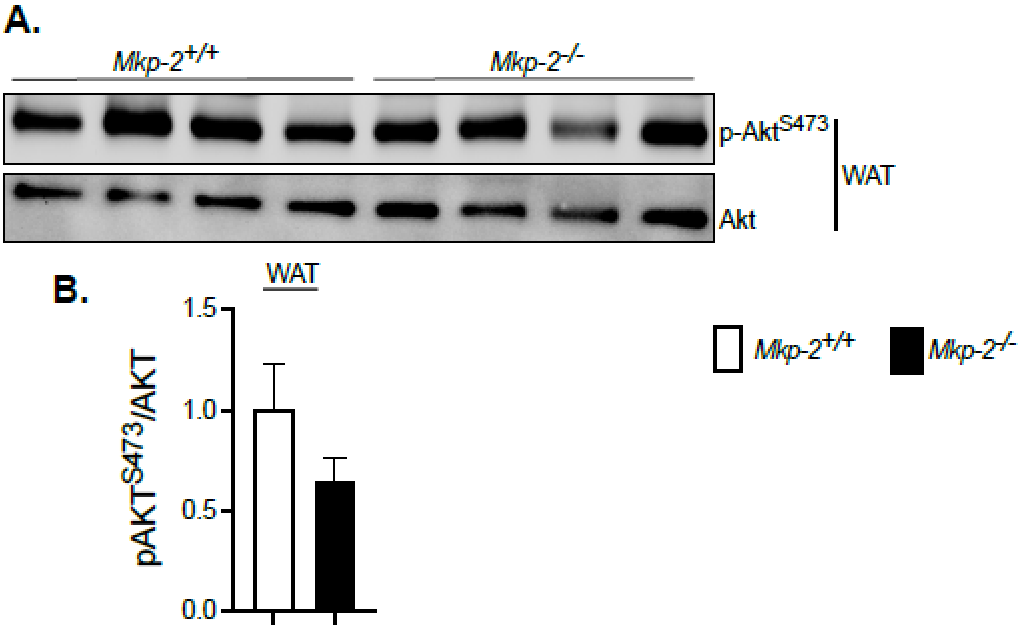

Figure S6. No difference in WAT Akt phosphorylation in MKP-2 deficient mice and wild type mice.

White adipose tissue lysates from overnight fasted insulin stimulated (i.p) HFD-fed (5 weeks) Mkp-$2^{+/+}$ and Mkp-$2^{-/-}$ mice were analyzed by immunoblotting (n=4-6mice/genotype). Representative immunoblots were quantitated by densitometry for phospho-Akt/Akt, liver. Results represent the mean ± SEM
